# Body Odor Disgust Sensitivity Predicts Moral Harshness Toward Moral Violations of Purity

**DOI:** 10.3389/fpsyg.2019.00458

**Published:** 2019-03-05

**Authors:** Marco Tullio Liuzza, Jonas K. Olofsson, Sebastian Cancino-Montecinos, Torun Lindholm

**Affiliations:** ^1^Department of Medical and Surgical Sciences, Università degli Studi Magna Græcia di Catanzaro, Catanzaro, Italy; ^2^Department of Psychology, Stockholm University, Stockholm, Sweden

**Keywords:** moral judgment, disgust, purity, behavioral immune system, body odors

## Abstract

Detecting pathogen threats and avoiding disease is fundamental to human survival. The behavioral immune system (BIS) framework outlines a set of psychological functions that may have evolved for this purpose. Disgust is a core emotion that plays a pivotal role in the BIS, as it activates the behavioral avoidance motives that prevent people from being in contact with pathogens. To date, there has been little agreement on how disgust sensitivity might underlie moral judgments. Here, we investigated moral violations of “purity” (assumed to elicit disgust) and violations of “harm” (assumed to elicit anger). We hypothesized that individual differences in BIS-related traits would be associated with greater disgust (vs. anger) reactivity to, and greater condemnation of Purity (vs. Harm) violations. The study was pre-registered (https://osf.io/57nm8/). Participants (*N* = 632) rated scenarios concerning moral wrongness or inappropriateness and regarding disgust and anger. To measure individual differences in the activation of the BIS, we used our recently developed Body Odor Disgust Scale (BODS), a BIS-related trait measure that assesses individual differences in feeling disgusted by body odors. In line with our predictions, we found that scores on the BODS relate more strongly to affective reactions to Purity, as compared to Harm, violations. In addition, BODS relates more strongly to Moral condemnation than to perceived Inappropriateness of an action, and to the condemnation of Purity violations as compared to Harm violations. These results suggest that the BIS is involved in moral judgment, although to some extent this role seems to be specific for violations of “moral purity,” a response that might be rooted in disease avoidance. Data and scripts to analyze the data are available on the Open Science Framework (OSF) repository: https://osf.io/tk4x5/. Planned analyses are available at https://osf.io/x6g3u/.

## Introduction

Detecting pathogen threats and avoiding disease is fundamental to human survival. The behavioral immune system (BIS, Schaller and Park, 2011) is a proposed set of psychological functions evolved to detect pathogen threats and avoid disease. Disgust is a core emotion that plays a pivotal role in the BIS, as disgust activates behavioral avoidance reactions which prevent people from being in contact with pathogens. For instance, people display higher levels of disgust when viewing pictures that convey the concept of contagion, as compared to physically similar pictures that do not convey any contagion-related concept (Curtis et al., 2004).

Are such intuitive avoidance reactions also relevant in making moral judgments on other people’s’ behaviors? Moral judgment was for a long time characterized as a result of “cold,” cognitive deliberation (Kohlberg, 1969). However, accumulating evidence supports instead a sentimentalist, or intuitionist, view (Haidt, 2001) that contends that affect may play a causal role in moral judgment. In fact, core – pathogen – disgust and moral disgust seem to be closely intertwined (Chapman et al., 2009; Vicario et al., 2018) and may share a common neurocognitive system (Vicario et al., 2017).

One method for testing this sentimentalist hypothesis is to induce feelings of disgust that, even though irrelevant, may amplify the severity of moral condemnation. Indeed, recent meta-analytical evidence suggests an amplification of the severity of moral condemnation when disgust is induced through taste or olfaction (Landy and Goodwin, 2015). Moreover, support for a causal link between feelings of disgust and moral judgments comes from recent research demonstrating that a chemical inhibition of nausea reduces the perceived severity of judgments toward moral violations (Tracy et al., 2019). These results are consistent with the notion that judgments of morality are associated with mechanisms evolved for detecting pathogen threats, and that the chemical senses (taste/olfaction) may be particularly relevant for this disgust-morality relation.

A pivotal question is whether incidental disgust is confined to the judgment of specific types of moral transgressions or to moral condemnation more generally. Graham et al. (2009) suggested that people’s morality rests on five moral foundations: harm/care, fairness/reciprocity, ingroup/loyalty, authority/respect, and purity/sanctity. In particular, purity principle violations (violations of decency norms) evoke disgust reactions (Graham et al., 2011) and high trait disgust is related to an emphasis on this moral foundation (van Leeuwen et al., 2017). Moreover, it was recently found that disgust sensitivity relates more strongly to moral condemnation of purity-based transgressions than to moral condemnation of transgressions in any of the other domains (Wagemans et al., 2018a). However, in their literature review on emotions and morality, Cameron et al. (2015), suggest that people experience as much disgust in response to purity transgressions as in response to harm transgressions. Hence, while some findings indicate that BIS-related emotions play a specific role in transgressions that have some ancestral relation with disease-related behaviors (e.g., sexually promiscuous behavior, Tybur et al., 2009), further evidence is needed to clarify this issue.

Another open issue is whether disgust plays a specific role in the amplification of moral condemnation, or whether negative emotions in general lead to such effects. Chapman and Anderson (2014) showed that disgust sensitivity, but not general emotionality (e.g., measured with STAI and trait aggression), predicts moral reactions, indicating that disgust has a uniquely moral function. Landy and Piazza (2017) in their Study 1 used the scenarios from Chapman and Anderson and, in addition, personal “imprudent actions,” namely actions where only the actor is affected by the behavior (“a person running in the rain or eating junk food”). Their results indicated that when reactivity in other emotions is tested with the same specificity as disgust [using the pathogen items from the *Three Domains of Disgust Scale* (TDDS), Tybur et al., 2009], several of these emotions are related to moral judgments as well. Moreover, they showed that more extreme condemnation of moral violations were not uniquely associated with disgust, but to emotional reactivity in general.

In the present study, we address these two open issues by examining reactions to a set of moral violation scenarios developed by Clifford et al. (2015). In their paper, Clifford and colleagues provide a standardized and validated set of moral violation scenarios that span across the five moral domains, with behaviors that violate a particular moral foundation and not others. For the current study, we chose scenarios that either depicted a violation of a care/harm moral principle, or a purity/sanctity moral principle (see section “Materials and Methods”). Participants rated the violations in terms of moral wrongness (“To what extent would you consider that the person’s behavior is morally wrong”) or inappropriateness (“How likely is it that you would show the person that the behavior is inappropriate?”) and in terms of disgust and anger elicited by that action. We hypothesized that purity violations should elicit stronger disgust, while harm violations should elicit stronger anger (Gutierrez and Giner-Sorolla, 2007).

Importantly, as a measure of disgust sensitivity related to the activation of the BIS, we used our recently developed Body Odor Disgust Sensitivity (BODS, Liuzza et al., 2016, 2017), a scale that assesses individual differences in feeling disgusted by body odors (e.g., “You are standing next to a stranger and notice that the t-shirt they are wearing smells strongly from their sweat”). Smells and tastes are arguably the most potent disgust signals, and it has been theorized that moral disgust is intimately linked to chemoreception (see Herz, 2012, for a review). Body-generated odors are considered strong disgust elicitors across cultures (Curtis and Biran, 2001), and are highly stigmatized in contemporary western culture (Soo and Stevenson, 2007). In fact, it has been argued that one of the most prominent functions of our sense of smell is to defend us from microbial hazards (Stevenson, 2009). Body odors can be affected by pathological processes (Shirasu and Touhara, 2011), and it has been shown that humans can detect the presence of an infection by the smell of the infected body (Olsson et al., 2014; Regenbogen et al., 2017). However, in established disgust scales, olfactory disgust in general, and body odor disgust in particular, occupies a very small space. We have previously established that scores on the BODS are strongly predictive of personality traits associated with harsher moral judgments, such as Right-Wing Authoritarianism (RWA), and that it is a better predictor of these traits than more general disgust sensitivity measures such as the TDDS (Liuzza et al., 2018). Informed by the theoretical link between body odor perception and disgust responses, and the evidence that the BODS is strongly predictive of traits related to moral condemnation, the current study explored whether the BODS, tapping into a core pathogen sensitivity, would help delineating the role of disgust in moral judgments.

We expected a moderating effect of the level of BODS on disgust responses. Specifically, we expected that people scoring higher in BODS would show a more pronounced Disgust reactivity (vs. Anger reactivity) to purity violations (vs. Harm violations). Similarly, we expected that people scoring higher in BODS would show harsher moral judgments concerning wrongness (vs. inappropriateness) to Purity violations (vs. Harm violations). Inappropriateness ratings were added in order to rule out that the condemnation of purity transgressions could be driven by the perception of weirdness (Gray and Keeney, 2015).

To rule out that the association between BODS and moral judgment could be fully explained by a general emotional reactivity, we tested whether our effects were retained when controlling for scores in the Emotional Reactivity Scale (ERS, Nock et al., 2008). Importantly, given the supposedly high relevance of the chemical senses in the experience of disgust (Rozin et al., 2009; Stevenson, 2009), and the linkage between incidental disgust and amplification/attenuation of moral condemnation (Landy and Goodwin, 2015; Tracy et al., 2019), we expected the BODS to provide incremental predictive validity when compared to general pathogen-related disgust sensitivity measures such as the pathogen subscale of the TDDS (TDDS-p, Tybur et al., 2009).

The BIS framework implies that psychological mechanisms adapted to detect and avoid pathogen threats may also encourage withdrawal from individuals who pose a threat to the group, such as members of unfamiliar out-groups or people who violate the established social order (e.g., Chapman and Anderson, 2014). As summarized in a recent review on the BIS (Ackerman et al., 2018) disgust leads to an overgeneralization of cues associated with disease (Makhanova et al., 2015) and prejudice toward unfamiliar outgroups (Faulkner et al., 2004; Zakrzewska et al., 2019). This connection between the BIS and avoidance-related social cognitions and behaviors may explain the consistent relation between BIS and social conservative attitudes (Terrizzi et al., 2013; Liuzza et al., 2018). Thus, according to this view, the relation between disgust and harshness in moral judgments should be mediated by individual differences in preference for the maintenance of social order. In support of this notion, recent research has demonstrated a robust association between disgust sensitivity and trait-level preference for orderliness, an association that, in turn, predicts political conservatism (Piazza and Sousa, 2014; Robinson et al., 2019). Considering the evidence suggesting that links from BIS-related traits to moral condemnation may be mediated by orderliness and social conservatism, we were interested in investigating the mediating role of these personality traits. Hence, we included a measure of orderliness, which is one of two aspects of trait conscientiousness (DeYoung et al., 2007), as well as the RWA scale. Therefore, we hypothesized that the moderation effect of the BODS on moral judgments should be mediated by individual differences in these traits.

We preregistered^1^ our hypotheses^2^, planned sample size, materials and methods and R scripts of the planned analysis on the Open Science Framework repository^3^.^4^

### Hypotheses

(1) We expected a two-way interaction with harmless moral violations (purity violations) evoking stronger disgust (vs. anger) as compared to harming moral violations. Such an effect, would confirm that disgust is specifically evoked by a subset of moral violations, rather than any type of moral violation.

(2) We expected a three-way interaction, with a moderating effect of the level in Body odor disgust sensitivity, as measured by the BODS scale (Liuzza et al., 2016) on disgust responses. Indeed, we expected that people scoring higher in BODS would show a more pronounced disgust-reactivity (vs. anger reactivity) to purity violations (vs. harm violations). This interaction would point toward a specific role of the individual differences in the BIS activation in reacting with disgust to body-related – but harmless – moral violations.

(3) We expected a three-way interaction, with a moderating effect of the level in BODS on moral wrongness (vs. inappropriateness) ratings. Specifically, we expected that people scoring higher in BODS would show harsher moral judgments in terms of wrongness (vs. inappropriateness) to purity violations (vs. harm violations). An interaction like this would point toward a specific role of the individual differences in the BIS activation in the moral condemnation of body-related – but harmless – moral violations.

(4) We hypothesized that, as compared to the Three Domains of Disgust Scale (Tybur et al., 2009), the BODS would show incremental validity by playing a stronger moderating role as compared to TDDS-p.

(5) We expected a main effect of the level of general emotional reactivity on affective reactions – higher affective ratings regardess of the emotion rated – but no interactions with the type of scenario or type of affective reaction.

(6) We hypothesized that the moderation effect of the BODS would be mediated by Orderliness, as measured by one aspect of trait conscientiousness within the Big Five Aspect Scale (BFAS; DeYoung et al., 2007) and Authoritarianism, as measured by the RWA (Zakrisson, 2005).

(7) We hypothesized that all the above effects hypotheses would remain significant when controlling for a general measure of Emotional Sensitivity as measured by the ERS (Nock et al., 2008). This effect would militate in favor of the idea that a specific BIS-related emotion (disgust), rather than a general emotional reactivity, that relates to the reaction to and the condemnation of body-related, harmless moral violations.

## Materials and Methods

### Ethics Statement

This research was conducted in full in accordance with the ethical principles outlined by the Swedish Research Council, http://www.codex.vr.se/, and with the 1964 Helsinki declaration and its later amendments. The current research did not include factors that require ethical vetting according to Swedish legislation on research ethics, http://www.epn.se/en/start/regulations/. All participants gave written informed consent before participating. Minimal risks studies are exempt from formal approval in the country where the study was conducted (Sweden).

### Participants

We had planned a sample of *n* = 620, which has a power of 80% to detect an effect as small as *r* = 0.1 having an alpha level = 0.05 and a planned directional hypothesis. Power analysis was performed in R Core Team (2018) through the “pwr” package (Champely, 2018). We recruited participants on Amazon Mechanical Turk (MTurk), for a compensation of 50¢. We recruited only participants with an approval rate of 85% and who participated in no fewer than 50 surveys and no more than 1,000 surveys. The lower limit served to provide a reliable estimate of participants’ reliability, while the upper limit aimed at limiting the participation of too-experienced MTurk workers.

Data were collected on Qualtrics between the 28th of November 2017 and the 3rd of December 2017. We excluded participants who had not completed the survey (i.e., marked as unfinished on MTurk). A final sample of 632 respondents participated in the study (279 Females, mean Age = 38.74, *SD* = 11.88). Of these participants, 0.96% had not graduated from high school, 9.63% were high school graduates, 23.92% had some college, 49.12% were college graduates, and 16.37% had a post-college degree.

### Measures

#### Body Odor Disgust Sensitivity

As a measure of body odor disgust sensitivity, we used the 12-items Body Odor Disgust Scale (BODS, Liuzza et al., 2016). BODS presents participants with a series of descriptions of situations (e.g., “You are standing next to a stranger and notice that the t-shirt they are wearing smells strongly from their sweat.”), and they have indicated the degree to which they find the situation disgusting on a five-point Likert-type item (1 = “Not disgusting at all” and 5 = “Extremely disgusting”).

#### Pathogen Disgust Sensitivity

As a measure of pathogen-related disgust sensitivity, we used the seven-items pathogen disgust scale from the Three Domain of Disgust Scale (Tybur et al., 2009). Pathogen disgust “is elicited by objects likely to contain infectious agents, including dead bodies, rotting foods, and bodily fluids such as feces, phlegm, vomit, blood, and semen, and it motivates proximal avoidance of such things” (Tybur et al., 2009, p. 105). To measure individual differences in pathogen disgust sensitivity, the participants rated how disgusting they would find each of the following concepts (e.g., “Accidentally touching a person’s bloody cut”) on a seven-point Likert-type item (0 = “Not disgusting at all” and 6 = “Extremely disgusting”).

#### Right-Wing Authoritarianism

In order to measure individual differences in RWA (Altemeyer, 1998), we used the Zakrisson’s RWA scale (Zakrisson, 2005), consisting of 15 items (e.g., “Our forefathers ought to be honored more for the way they have built our society, at the same time we ought to put an end to those forces destroying it.”) that did not refer to specific minority populations, and hence avoided conflating authoritarianism with specific prejudice. Participants reported their reaction to each statement on 15 seven-point Likert-type items (1 = “Very negative” and 7 = “Very positive”).

#### Emotional Reactivity Scale

We used the ERS to measure individual differences in general reactivity. The ERS is a 21-item self-report measure designed to assess individuals’ everyday emotional experiences. Participants are asked to state on a five-point scale (0 = not at all like me; 1 = a little like me; 2 = somewhat like me; 3 = a lot like me; 4 = completely like me) to what extent different statements regarding emotional experiences characterizes them. Example items are: “I experience emotions very strongly”; “If I have a disagreement with someone, it takes a long time for me to get over it”; “I am often bothered by things that other people don’t react to.” The ERS has been found to be related to depressive mood, frustration, aggression, fear, and shyness. Furthermore, the scale is inversely related to attentional control, inhibitory control, and activation control. Thus, the ERS is a broad and general measure of emotional reactivity (see e.g., Nock et al., 2008).

#### Orderliness

Trait orderliness is one of two aspects of trait conscientiousness (the other is industriousness) identified within the Big Five Aspects Scales (DeYoung et al., 2007). Orderliness is characterized by a general predisposition toward maintaining structure, organization, and neatness. On the 10 items assessing orderliness, participants rate their level of agreement with statements such as “I want every detail taken care of.”

#### Affective Reactions and Evaluative Judgments to Moral Violation Scenarios

We used eight scenarios from the material developed by Clifford et al. (2015), a standardized and validated set of moral transgressions that encompasses the moral domains as proposed by the Moral Foundations Theory. Four scenarios violated moral norms concerning Care/Harm, and four violated norms concerning Purity/Sanctity. The four Care/Harm scenarios were selected to represent different facets of the this principle: (i) emotional harm (“You see a teenage boy chuckling at an amputee he passes by while on the subway”), physical harm directed toward (ii) animals (e.g., “You see a woman throwing her cat across the room for scratching the furniture,”) (iii) humans (“You see a teacher hitting a student’s hand with a ruler for falling asleep in class”). The selected purity scenarios reflected different aspects of this principle (i) sexually deviant acts (“You see a man having sex with a frozen chicken before cooking it for dinner,”) (ii) contamination concerns (“You see a woman having intimate relations with a recently deceased loved one”), (iii) degrading (“You see a man searching through the trash to find women’s discarded underwear”). Participants then rated on seven-point Likert-type (1 = “Not at all” and 7 = “To a very high extent”) items:

(a) to what extent they would feel (i) *anger* (Anger Rated Emotion) and (ii) *disgust* (Disgust Rated Emotion).
(b) to what extent they would consider that the person’s behavior was (i) morally wrong (Moral Wrongness Moral Rating of condemnation) (ii) how likely they would show the person that the behavior is inappropriate (1 = “Not likely,” 7 = “Most likely,” Inappropriateness Moral Rating of condemnation).

All the conditions were manipulated within subjects. Hence, each participant read the eight scenarios, and rated them in terms of evoked anger/disgust, and inappropriateness/moral wrongness.

### Data Analysis

Prior to computing the scores for our measures, we tested our measures’ dimensionality and reliability. Failure to achieve an acceptable level of internal consistency (Cronbach’ α ≥ 0.6), and/or failure to achieve an acceptable goodness of fit for assumptions of uni-dimensionality (RMSEA > 0.1, SRMR > 0.1, CFI < 0.90, TLI < 0.90) led to further inspection in order to exclude the items that impede to reach an acceptable reliability and/or uni-dimensionality. Dimensionality was tested using the *cfa* function from the *lavaan* package in R (Rosseel, 2012), while Cronbach’s α was assessed using the *alpha* function from the *psych* package in R (Revelle, 2018).

For each variable, we computed the mean value and standardized it, except for the affective and the moral ratings provided for each scenario, that were only standardized in order to not lose information about the sources of variability, while accounting for the dependence of these observations. We started with a zero-order correlation matrix, then we tested the hypotheses, by conducting a linear multilevel model (LMM or “mixed effects models”; Pinheiro and Bates, 2000), through the package lme4 ver. 1.1–5 (Bates et al., 2015). We used the *Anova* function from the *car* package (Fox and Weisberg, 2011) to compute the χ^2^ associated with the Type III Walden test. Contrasts were set through an effect coding strategy (e.g., [1, -1]), in order to better interpret the main effects and interactions. The first model tested our hypotheses on anger and disgust ratings:

(1) If the type of scenario (purity vs. harm) evokes a stronger disgust (vs. anger) reaction to that scenario.
(2) If the BODS interacts with the type of scenario (moral violations of purity vs. harm) to predict disgust (vs. anger).
(3) Then, in a second and third model, we added the TDDS-p and the ERS to the model as moderators along with the BODS.
(4) We used identical models to test ratings of moral wrongness vs. inappropriateness.

## Results

### Measures of Reliability and Dimensionality

#### Body Odor Disgust Scale

An initial assessment of the BODS dimensionality as a unidimensional construct showed very poor fit in the present dataset (RMSEA = 0.22, SRMR = 0.09, TLI = 0.68, CFI = 0.74).

We thus tried a two-factor solution (Internal Body Odor Source and External Body Odor source), consistent with a prior validation study (Liuzza et al., 2016), and allowed covariance of the residuals within each odor type. These changes led to an acceptable fit (RMSEA = 0.1, SRMR = 0.04, TLI = 0.93, CFI = 0.95). However, the two latent variables were highly correlated (*r* = 0.72). Therefore, any model using them as separate predictors would have posed issues of collinearity. A CFA assuming a hierarchical structure with the two subscales (Internal, External) as emanating from the same underlying BODS factor showed an acceptable fit (RMSEA = 0.1, SRMR = 0.04, TLI = 0.93, CFI = 0.95) and was consistent with our theoretical assumptions. We therefore continued our analyses by treating the BODS as a unique scale. The internal consistency of the two subscales was excellent (Cronbach’s α > 0.9) and so it was the internal consistency of the scale when considered as a unique scale (Cronbach’s α = 0.94).

#### Three Domains of Disgust – Pathogen Subscale

The pathogen sub-scale of the TDDS, on the other hand, immediately showed an acceptable fit when using a unidimensional model (RMSEA = 0.1, SRMR = 0.04, TLI = 0.92, CFI = 0.95). We thus treated TDDS-p as a unidimensional measure and the scale internal consistency was good (Cronbach’s α = 0.85).

#### Emotional Reactivity Scale

CFA on the ERS showed a poor fit for the unidimensional model (RMSEA = 0.13, SRMR = 0.07, TLI = 0.8, CFI = 0.82). An exploration of the modification indices showed a high level of covariation between some items’ residuals, possibly because of substantial semantic overlap. After allowing for covariance between some of the items’ residuals, the model nearly reached an acceptable fit (RMSEA = 0.1, SRMR = 0.06, TLI = 0.89, CFI = 0.91). We thus treated ERS as a unidimensional measure and the scale showed excellent internal consistency (Cronbach’s α = 0.96).

#### Right-Wing Authoritarianism

The RWA did not show an acceptable fit when using a unidimensional model (RMSEA = 0.15, SRMR = 0.09, TLI = 0.75, CFI = 0.78). After looking at the modification indices, we decided to allow for covariation between the residuals of some items. After this modification, the RWA achieved an acceptable fit (RMSEA = 0.09, SRMR = 0.06, TLI = 0.91, CFI = 0.93), and the scale internal consistency was excellent (Cronbach’s α = 0.93).

#### Orderliness

The Orderliness scale did not show an acceptable fit when using a unidimensional model (RMSEA = 0.14, SRMR = 0.08, TLI = 0.76, CFI = 0.81). After looking at the modification indices, we decided to allow for covariation cross the residuals of some items. In most cases, this covariation was among items that were straight coded items. After this modification, the Orderliness scale achieved an acceptable fit (RMSEA = 0.08, SRMR = 0.05, TLI = 0.91, CFI = 0.94), and the scale internal consistency was good (Cronbach’s α = 0.85).

### Descriptive Statistics and Zero-Order Correlations

Table 1 provides the descriptive statistics or our measures. Although not planned in advance, we also computed the internal consistency for the affective and moral ratings, and found that, for each type of rating in each condition, the average inter-item correlation ranged between *r* = 0.41 (Moral wrongness in the Harm Condition) to *r* = 0.60 (Disgust in the Purity Condition).

**Table 1:** Descriptive statistics.

	Mean	*SD*	Median	Min	Max	Skewness	Kurtosis
Harm Anger	5.36	1.37	5.50	1	7	–0.87	0.32
Harm Disgust	5.03	1.52	5.25	1	7	–0.74	–0.07
Purity Anger	4.22	1.84	4.25	1	7	–0.20	–1.11
Purity Disgust	6.02	1.28	6.50	1	7	–1.67	2.33
Harm Moral	5.63	1.22	5.75	1	7	–1.05	0.94
Harm Inapp	5.18	1.53	5.50	1	7	–0.80	0.00
Purity Moral	5.45	1.47	5.75	1	7	–0.97	0.19
Purity Inapp	4.81	1.88	5.00	1	7	–0.54	–0.85
BODS	3.57	0.84	3.58	1	5	–0.40	–0.18
Reac	2.63	0.95	2.57	1	5	0.21	–0.78
TDDS	4.81	1.16	4.86	1	7	–0.30	–0.02
RWA	3.25	1.33	3.40	1	6.73	0.11	–0.62
ORD	3.63	0.72	3.70	1.6	5	–0.03	–0.55


*Descriptive statistics for the measures and for the ratings on Disgust, Anger, Moral wrongness (Moral) and Inappropriateness (Inapp) evoked by Harm and Purity Scenarios. SD, standard deviation; BODS, Body Odor Disgust Scale score; ERS, Emotional Reactivity; TDDS-p, Three Domains of Disgust – pathogen subscale; RWA, Right Wing Authoritarianism; ORD, orderliness.*

Table 2 shows the zero-order correlations. Replicating previous results (Liuzza et al., 2018), we found a moderate (*r* = 0.25), but statistically significant (*p* < 0.001) correlation between the RWA scores and the BODS. The BODS was positively and significantly associated, to various degrees, to all the other measures. It is worth noticing that the very high level of correlation with the TDDS-p (*r* = 0.69) may undermine the interpretability of the results from models in which both variables are included, due to multicollinearity issues.

**Table 2:** Zero-order correlations across measures.

Variable	1	2	3	4	5
(1) BODS					
(2) ERS	0.16^∗∗∗^ [0.09, 0.24]				
(3) TDDS-p	0.69^∗∗∗^ [0.64, 0.73]	0.21^∗∗∗^ [0.13, 0.28]			
(4) RWA	0.25^∗∗∗^ [0.17, 0.32]	0.10^∗∗^ [0.03, 0.18]	0.20^∗∗∗^ [0.12, 0.27]		
(5) ORD	0.26^∗∗∗^ [0.18, 0.33]	–0.07 [-0.14, 0.01]	0.28^∗∗∗^ [0.20, 0.35]	0.18^∗∗^ [0.10, 0.26]	
(6) Age	0.12^∗∗^ [0.04, 0.20]	–0.20^∗∗∗^ [-0.27, -0.12]	0.03 [-0.05, 0.11]	0.06 [-0.02, 0.14]	0.12^∗∗^ [0.04, 0.20]


*BODS, Body Odor Disgust Scale score; ERS, Emotional Reactivity; TDDS-p, Three Domains of Disgust – pathogen subscale; RWA, Right Wing Authoritarianism; ORD, orderliness. M and SD are used to represent mean and standard deviation, respectively. Values in square brackets indicate the 95% confidence interval for each correlation. ^∗^*p* < *0*.05; ^∗∗^*p* < *0*.01***;***^∗∗∗^*p* < *0*.001.*

Zero-order correlations between the BODS and the ratings ranged between *r* = 0.21 (Inappropriateness in the Purity Condition) to *r* = 0.39 (Disgust, and Moral wrongness in the Purity Condition). All the correlations were statistically significant (*p*s < 0.001).

### Gender Differences

We also checked for gender difference through a series of unpaired *t*-tests. We found that women displayed significantly higher levels of BODS scores, TDDS-p scores, and orderliness scores [*t*s(619) > 2.83, *p*s < 0.01], a finding that replicates previous research (e.g., Mancini et al., 2001; Tybur et al., 2009; Liuzza et al., 2016), although the size of these effects are small (Cohen’s Ds < = 0.37). No gender difference was found in RWA scores [*t*(619) = 0.19, *p* > 0.05, Cohen’s D = -0.02].

### Emotions

In order to test our Hypothesis 1 we fit an LMM with the rating in the affective dimension as our dependent variable and we entered the main effects and all the possible interactions between the rated emotion (disgust vs. anger), the type of moral violation (purity vs. harm), and the scores in the BODS. We added Age, Gender, and Education as covariates to adjust for. Random intercepts for participants were included in the model. However, the random slopes were not included because adding random slopes would have led to convergence issues due to the small number of repetitions per condition (ranging from 4 to 8). This choice is coherent with the pre-registered analysis when we observed convergence issues even in the simulations prior to the launch of the study.

**FIGURE 1:**
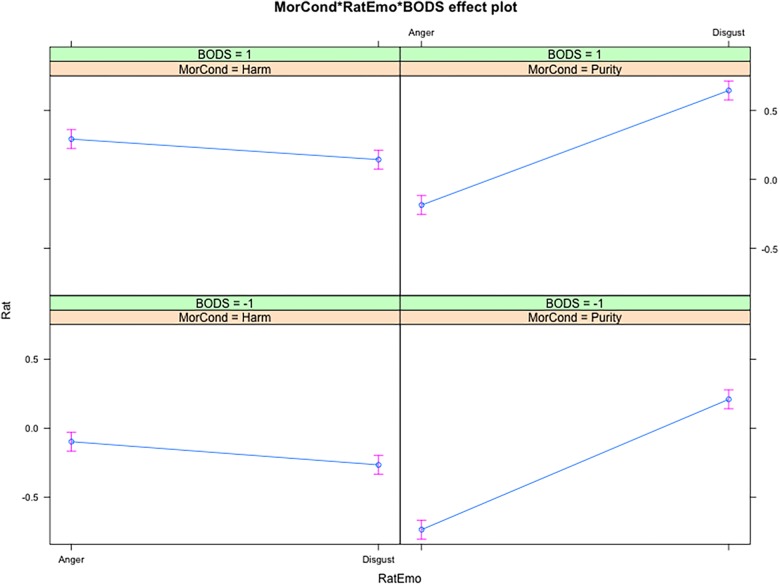
Interaction effects of BODS, Moral Condition and Rated Emotion on affective ratings. Results are displayed for levels of BODS = –1 SD (upper panels) and BODS = +1 SD (lower panels). MorCond, Moral Condition (Harm vs. Purity); RatEmo, Rated Emotion (Disgust vs. Anger); BODS, Body Odor Disgust Scale score.

As Table 3 shows, we found a main effect of participant gender on affective ratings, with women providing higher ratings than men (β = 0.19, *SE* = 0.02). The main effect of Moral Condition (Purity vs. Harm scenarios) was explained by higher overall affective reactions to the harm violations, as compared to the purity violations (β = 0.02, *SE* = 0.01), while the effect of the Rated Emotion (anger vs. disgust) was explained by the higher overall ratings in disgust, as compared to anger (β = -0.18, *SE* = 0.01). Importantly, the two effects were qualified by our predicted two-way interaction (Hypothesis 1) between Moral Condition and Rated Emotion, with Purity transgressions evoking stronger Disgust (vs. Anger) than Harm transgressions. In addition, we found a main effect of BODS in predicting Rated Emotion, such that higher BODS scores were associated with higher ratings of both disgust and anger (β = 0.22, *SE* = 0.02). This main effect was implicit in our assumptions, although not explicitly stated in our hypotheses where we focused on interactions between the BODS and other variables. Importantly, we found a significant interaction, anticipated in our Hypothesis 2, with a moderating effect of BODS scores on disgust responses in interaction with the scenario (see Figure 1). However, in contrast to our expectations, people scoring higher as compared to low in BODS did not display a more pronounced Disgust reactivity (vs. Anger reactivity) to Purity violations (vs. Harm violations). Follow-up analysis on the two-way interaction in the two different moral scenarios showed that for the Harm violations, the slope for the relation between BODS and Anger ratings was not significantly different from the slope for the relation between BODS and Disgust ratings (β = 0, *SE* = 0.01, p > 0.05). On the other hand, in the Purity condition, the slope for the relation between BODS and Anger ratings was significantly different from the slope for the relation between BODS and Disgust ratings (β = 0.03, *SE* = 0.01, p < 0.01). In other words, in the Purity condition, the difference between participants scoring high vs. low in BODS was higher for the Anger than for the Disgust ratings (see Figure 1, right panels).

**Table 3:** Type III Wald χ**^2^** Analysis of Deviance on affective ratings on moral scenarios.

	χ^2^	Df	*p*
Age	0.92	1	0.338
**Gender**	**81.39**	**1**	**<0.001**
Education	0.16	1	0.693
**MorCond**	**5.29**	**1**	**0.021**
**RatEmo**	**587.56**	**1**	**<0.001**
**BODS**	**111.24**	**1**	**<0.001**
**MorCond** ×**RatEmo**	**1210.44**	**1**	**<0.001**
**MorCond** ×**BODS**	**9.48**	**1**	**0.002**
RatEmo × BODS	2.47	1	0.116
**MorCond** ×**RatEmo** ×**BODS**	**4.96**	**1**	**0.026**


*MorCond, Moral Condition (Harm vs. Purity); RatEmo, Rated Emotion (Disgust vs. Anger); BODS, Body Odor Disgust Scale score. Bold text indicates a statistically significant correlation with a *p*-value less than 0.05.*

To test our Hypothesis 4, we added TDDS-p to our model as the main effect and as a moderator. Table 4 shows that adding the TDDS-p reduces the interaction between BODS, Moral Condition, and the Rated Emotion to non-significance. Instead, the analysis shows an interaction between TDDS-p and Moral scenario that approaches significance (*p* = 0.055), and that mirrors the moderating effects of the BODS. Thus, TDDS-p tended to affect ratings of Anger rather than Disgust in the Purity condition. This result disconfirms the predictions we made in Hypothesis 4 and rather suggests that, when it comes to moral violations, TDDS-p seems to have a better incremental validity than BODS. It should be observed, however, that the correlation between TDDS-p and the BODS found in this study is so high (Pearson’s *r* = 0.69) that it undermines the interpretability our results, given the high level of multicollinearity.

**Table 4:** Type III Wald χ^2^ Analysis of Deviance on affective ratings on moral scenarios.

	χ*^2^*	Df	*p*
Age	2.34	1	0.126
**Gender**	**70.73**	**1**	**<0.001**
Education	0.24	1	0.624
**MorCond**	**5.34**	**1**	**0.021**
**RatEmo**	**588.45**	**1**	**<0.001**
**BODS**	**11.30**	**1**	**0.001**
**TDDS-p**	**45.07**	**1**	**<0.001**
**MorCond** ×**RatEmo**	**1212.26**	**1**	**<0.001**
MorCond × BODS	0.07	1	0.792
RatEmo × BODS	0.04	1	0.850
**MorCond** ×**TDDS-p**	**8.13**	**1**	**0.004**
**RatEmo** ×**TDDS-p**	1.90	1	0.169
**MorCond** ×**RatEmo** ×**BODS**	0.08	1	0.771
**MorCond** ×**RatEmo** ×**TDDS-p**	3.67	1	0.055


*MorCond, Moral Condition (Harm vs. Purity); RatEmo, Rated Emotion (disgust vs. anger); BODS, Body Odor Disgust Scale score; TDDS-p, Three Domains of Disgust, pathogen scale. Bold text indicates a statistically significant correlation with a *p*-value less than 0.05.*

Importantly, the main effect of the BODS on the Disgust and Anger ratings is still significant after including TDDS-p (β = 0.09, *SE* = 0.028, *p* = 0.001). This suggests that, at least for the main effect, there is substantial shared variance between BODS and affective reactions to moral scenarios that is independent of the shared variance between TDDS-p and affective reactions.

In order to test our Hypothesis 5, we entered ERS scores into our model. We found a main effect of ERS on affective ratings, in line with our hypothesis (Table 5).

**Table 5:** Type III Wald χ^2^ Analysis of Deviance on affective ratings on moral scenarios.

	χ^2^	Df	*p*
Age	2.52	1	0.112
**Gender**	**73.07**	**1**	**<0.001**
Education	0.05	1	0.828
**MorCond**	**5.37**	**1**	**0.020**
**RatEmo**	**590.81**	**1**	**<0.001**
**BODS**	**99.31**	**1**	**<0.001**
**ERS**	**8.22**	**1**	**0.004**
**MorCond** ×**RatEmo**	**1217.09**	**1**	**<0.001**
**MorCond** ×**BODS**	**13.03**	**1**	**<0.001**
RatEmo × BODS	0.81	1	0.368
**MorCond** ×**ERS**	**11.80**	**1**	**0.001**
**RatEmo** ×**ERS**	**16.05**	**1**	**<0.001**
MorCond × RatEmo × BODS	1.50	1	0.221
**MorCond** ×**RatEmo** ×**ERS**	**35.80**	**1**	**0.001**


*MorCond, Moral Condition (Harm vs. Purity); RatEmo, Rated Emotion (Disgust vs. Anger); BODS, Body Odor Disgust Scale score; ERS, Emotional Reactivity. Bold text indicates a statistically significant correlation with a *p*-value less than 0.05.*

**FIGURE 2:**
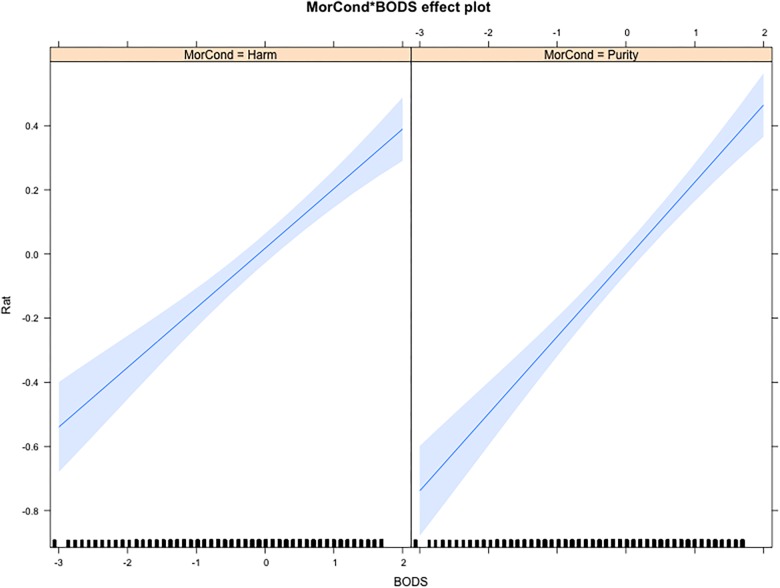
Interaction effect of BODS and Moral Condition on affective ratings, when controlling for overall Emotion Reactivity. MorCond, Moral Condition (Harm vs. Purity); BODS, Body Odor Disgust Scale score.

However, the only interaction that included BODS and remained significant when controlling for general Emotional Reactivity was the one between the BODS and Moral Condition, as described earlier (see Figure 2).

Thus, when adding the ERS, the association between the BODS and the intensity of the Disgust and Anger was still stronger in the purity scenario than in the harm scenario (β = -0.03, *SE* = 0.008). However, contrary to our Hypothesis 5, ERS displayed a significant triple interaction with moral scenarios and type of rated emotion. Interestingly, ERS is positively associated with both Anger and Disgust ratings in the Harm condition, and with Anger ratings in the Purity condition (βs ≥ 0.1, 0.02 > *SE* < 0.04), but displays a null pattern of association with Disgust ratings in the Purity condition (β = 0, *SE =* 0.03). Since the Purity scenarios seem to evoke higher disgust ratings even among low ERS participants, this result might be due to ceiling effects (see Table 1).

In partial coherence with what anticipated in our Hypothesis 7, the findings on the BODS are still significant when controlling for ERS. Hypothesis 6 was not further tested because we could not reject the null hypothesis for Hypothesis 2.

### Moral Judgment

In order to test our Hypothesis 3, we ran the same analysis as for Hypothesis 2 but on moral ratings (Inappropriateness vs. Moral Wrongness).

**FIGURE 3:**
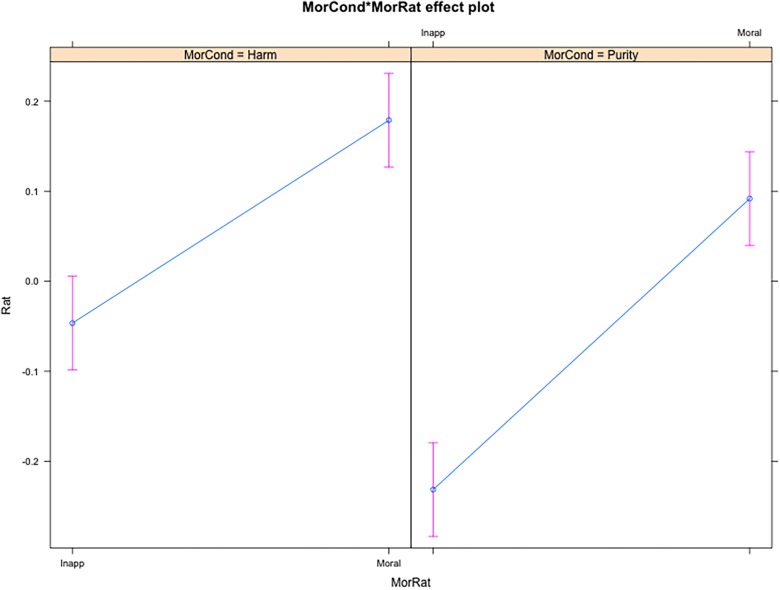
Interaction effect of Moral Condition and Moral Ratings on condemnation ratings. MorCond, Moral Condition (Harm vs. Purity); MorRat, Moral Rating; Moral, Morally Wrong; Inapp, inappropriate.

Results from moral ratings were similar to those from affective ratings (see Table 6), with a main effect of the BODS on both moral ratings. We found an interaction between the Moral Condition and Moral Rating such that participants reported more willingness to call a behavior as Inappropriate (vs. Wrong) in Harm violation scenarios (vs. Purity, see Figure 3).

**Table 6:** Type III Wald χ^2^ Analysis of Deviance on Moral judgments.

	χ^2^	Df	*p*
Age	0.00	1	0.957
**Gender**	**27.83**	**1**	**<0.001**
Education	0.91	1	0.339
**MorCond**	**71.97**	**1**	**<0.001**
**MorRat**	**293.05**	**1**	**<0.001**
**BODS**	**73.03**	**1**	**<0.001**
**MorCond** ×**MorRat**	**9.32**	**1**	**0.002**
**MorCond** ×**BODS**	**11.00**	**1**	**0.001**
**MorRat** ×**BODS**	**15.93**	**1**	**<0.001**
MorCond × MorRat × BODS	2.38	1	0.123


*MorCond, Moral Condition (Harm vs. Purity); MorRat, Moral Rating (Wrongness vs. Inappropriateness); BODS, Body Odor Disgust Scale score. Bold text indicates a statistically significant correlation with a *p*-value less than 0.05.*

**FIGURE 4:**
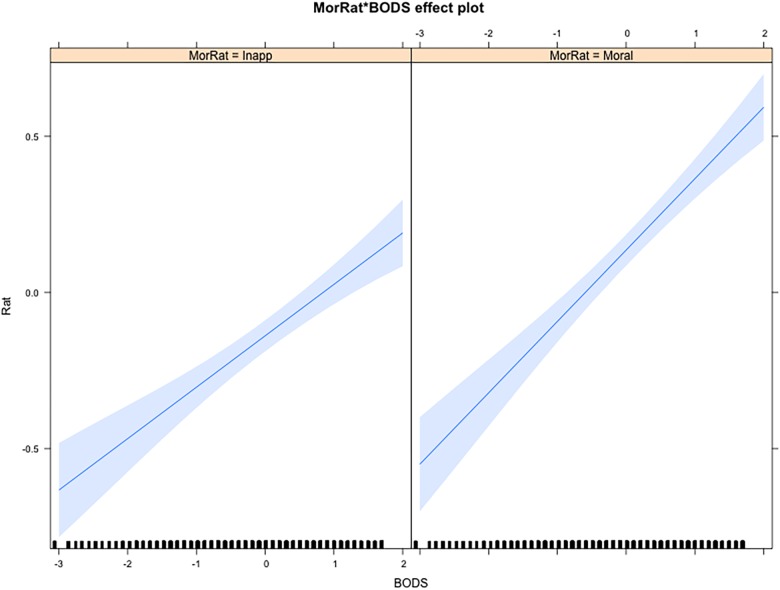
Interaction effect of BODS scores and Moral Ratings on condemnation ratings. BODS, Body Odor Disgust Scale; MorRat, Moral Rating; Moral, Morally Wrong; Inapp, inappropriate.

As was the case for the affective ratings, the BODS holds a stronger association with moral judgments – regardless of the type – in the Purity condition, as opposed to the Harm condition (β = 0.03, *SE* = 0.008). This interaction remained significant even when controlling for ERS (β = 0.03, *SE* = 0.008, *p* < 0.001) but not TDDS-p (β = -0.01, *SE* = 0.011, *p* > 0.05). Furthermore, the BODS holds a stronger association with the Moral Wrongness ratings than the Inappropriateness ratings (β = 0.03, *SE* = 0.008, Figure 4). This interaction remained significant even when controlling for TDDS-p (β = 0.02, *SE* = 0.011, *p* = 0.033) and ERS (β = 0.03, *SE* = 0.008, *p* < 0.001). However, the predicted three-way interaction, with a moderating effect of the level in the BODS on Moral Wrongness (vs. Inappropriateness) in the Purity (vs. Harm) condition was not significant (*p* > 0.05).

## Discussion

Individual differences in traits that are related to the BIS are also consistently related to moral condemnation (for a review, see Russell and Giner-Sorolla, 2013). Disgust sensitivity, and disgust sensitivity to body odors in particular, plays a central role in the BIS (Liuzza et al., 2016). However, the role of disgust in moral judgment is controversial (Schnall et al., 2008; Chapman et al., 2009; Cameron et al., 2015; Landy and Goodwin, 2015; Wagemans et al., 2018a,b). We hypothesized that a novel body odor disgust sensitivity assessment would tap into a core pathogen sensitivity and help explaining moral judgments (BODS, Liuzza et al., 2016, 2017). Specifically we asked whether (a) disgust is evoked by purity violations to a greater extent than anger, (b) individual differences in the BODS moderate this effect, (c) higher scores in BODS predict harsher moral condemnation.

Before specific results are discussed, some possible limitations to the generalizability of the current results should be considered. In this study, we recruited a sample from a Mechanical Turk (M-Turk) pool. Firstly, it is well-known that this kind of sample is not representative of the population. In fact, M-Turk pools are self-selected samples, which may differ from the population in some important demographic and political features (Berinsky et al., 2012; Huff and Tingley, 2015). However, M-Turk samples have been shown to be more diverse and representative than common convenience samples (e.g., college students) typically used in psychological research (Buhrmester et al., 2011; Berinsky et al., 2012). Also important, M-Turk studies have been shown to be psychometrically valid (Buhrmester et al., 2011; Shapiro et al., 2013). Secondly, M-Turk workers may have participated in studies, and therefore they might not be naive to the purpose of the study. This may lead to biases due to participants’ expectations. Recent findings suggest that non-naivetè does not affect the performance in cognitive tasks (Zwaan et al., 2017), however, we cannot rule out that this is the case in other domains such as Moral Psychology.

Another limitation that concerns the validity of our measure of disgust sensitivity is the observation that so far we have measured disgust sensitivity using a self-report measure that may not be so predictive of the actual behavioral response to disgusting cues. Although we do have some evidence in favor of the criterion validity of our measure (Liuzza et al., 2017), it would be preferable to run a study in which participants are actually exposed to disgusting odors (e.g., Inbar et al., 2012; Cecchetto et al., 2017). In fact, so far we are unsure whether our results reflect genuine differences in the sensitivity to disgusting stimuli, or rather differences, for instance, in seeing disgust sensitivity as an appealing trait.

In the current study, we found support for our hypothesis that Purity transgressions (e.g., “You see an employee at a morgue eating his pepperoni pizza off of a dead body”), as compared to Harm transgression (“You see a teacher hitting a student’s hand with a ruler for falling asleep in class.”), elicited a greater Disgust reaction, as compared to Anger. This finding fits well with those of Wagemans et al. (2018a), who investigated effects of trait anger and trait disgust on moral judgments. Our findings, however, extend their results to situation-specific affective reactions.

Although we expected that people scoring higher in BODS would show a more pronounced Disgust reactivity (vs. Anger reactivity) to purity violations (vs. harm violations), the Purity condition instead revealed a stronger relationship between BODS and anger ratings, compared to disgust ratings. A closer look at the results, however, suggests that the interaction might be artificially determined by ceiling effects in the disgust ratings for purity violations among people high in BODS (see Figure 1, and the results of the ERS × Moral Condition × Rated Emotion interaction). In other words, it appears that people scoring high in the BODS already provided nearly maximal ratings of disgust in the Purity condition, and therefore there was not enough leverage for providing even higher ratings. The failure to find the expected moderation role of the BODS on the interaction between disgust and Moral Condition might therefore due to limitations concerning the measurement of this BIS-related underlying construct. Alternatively, our negative finding might be partially consistent with the results from Landy and Piazza (2017), who failed to find a stronger relationship between trait disgust and moral condemnation, as compared to other negative emotions.

Nonetheless, we found a stronger association between BODS and affective reactivity (regardless of the specific emotion) in the purity violations scenarios, as compared to the association observed in the harm violations scenarios. This effect remained significant even when controlling for general emotional reactivity. This finding is consistent with the idea that people who exhibit higher levels of trait disgust may react more strongly to moral violations in general (Jones and Fitness, 2008), and that disgust prompts stronger reactions to moral violations (Rozin and Fallon, 1987; Rozin et al., 2009; Tybur et al., 2013) in accordance with a neo-sentimentalist stance on moral judgment (Haidt, 2001).

In order to gain specificity and differentiate moral condemnation from social appropriateness, we collected ratings on both dimensions. Gray and Keeney (2015) have suggested that what differentiates the Purity scenarios from the Harm ones is not their more intimate relation with disgust, but rather their weirdness (e.g., having sex with a frozen chicken). Wagemans et al. (2018a), however, did not find any association between sensitivity to deviation (Okimoto and Gromet, 2016) and moral condemnation. In a study specifically designed to test whether weirdness of disgust sensitivity items predict their relationship to moral judgments of purity transgressions, Wagemans et al. (2018b) found that eliminating the weirdest items from disgust sensitivity measures did not eliminate the more pronounced association between disgust sensitivity and moral judgments for purity transgressions. In addition, our results show that actually, our participants rated the Purity violations less – not more – inappropriate/weird than the Harm violations (Figure 3). Furthermore, we found that the BODS scores are more strongly associated with Moral Wrongness ratings than Inappropriateness (Figure 4). Hence, it seems unlikely that the weirdness of Purity transgression explains the higher condemnation among people with higher levels of BIS-related traits. However, it should be noted that the validity of the inappropriateness rating to measure weirdness may be questioned. In fact, some participants may interpret inappropriateness as a synonym of moral wrongness. This potential limitation cannot be ruled out in this study.

In the current study, we included only the TDDS pathogen subscale as a control (TDDS-p, e.g., ‘Sitting next to someone who has red sores on their arm’), in order to provide the closest and most relevant comparison to body odor disgust. Subscales measuring sexual disgust (e.g., ‘Watching a pornographic video’) and moral disgust (e.g., ‘A student cheating to get good grades’) were not included because they are trivially related to moral judgment. Therefore, controlling for them would have undermined our inference because we would have controlled for a mediator (Rohrer, 2018).

Although the current study has the strength of being well-powered and pre-registered, the results were not as straightforward as expected. For instance, although the effect of disgust sensitivity on emotional reactivity to violations appears to be stronger in the Purity (vs. Harm) Condition, we did not find the effect to be specific to disgust ratings. Moreover, whereas the BODS scores were more strongly related to a Moral wrongness judgment (vs. Inappropriateness), this effect did not interact with the Moral scenario.

The inconclusiveness of our findings might be related to the some limitations in our measures of disgust sensitivity. In fact, when measuring disgust sensitivity through supposedly disgust evoking items, other emotions might be evoked as well. In the future, we may ask participants to rate also how much each of the BODS items evoke other negative emotions, following the same strategy as by Landy and Piazza (2017). This might provide a more fine-grained picture on the supposed selective relationship between disgust and moral condemnation of specific moral violations related to the preservation of purity.

Overall, our data suggest that body odor disgust sensitivity (part of the BIS) might in part explain affective reactions to a victimless moral violation that threatens the moral foundation of purity. Such a link might be expected because responses to purity violations have been linked to disease-avoidance concerns (Haidt and Graham, 2007). However, because of the lack of specificity in terms of elicited emotion (Disgust vs. Anger), we cannot rule out the hypothesis that individual differences in disgust sensitivity may covary with a greater tendency to provide extreme ratings. Such a finding might be in line with what found by Landy and Piazza (2017). The fact that this relationship appears to be stronger in response to Purity violations, is in line with the observation that these violations elicit a more automatic, less flexible response (Russell and Giner-Sorolla, 2011; Sabo and Giner-Sorolla, 2017).

## Conclusion

Individuals with high body odor disgust sensitivity experience stronger affective reactions to moral violations, especially when Purity principles are violated, and strongly condemn morally deviant actions, suggesting a link between chemosensory functions and emotions driving moral judgments of the behaviors of others.

## Data Availability

Data associated with this research are available on the Open Science Framework at https://osf.io/tk4x5/.

## Author Contributions

MTL, TL, SC-M, and JO designed the research. MTL and TL conducted the research. MTL analyzed the data. MTL, TL, and JO wrote the manuscript. All authors approved the final version of the manuscript.

## Conflict of Interest Statement

The authors declare that the research was conducted in the absence of any commercial or financial relationships that could be construed as a potential conflict of interest.

## Footnotes

1 Due to a misunderstanding in the pre-registration procedure, our documents were saved as a draft, but not published, on the 28th of November 2017. We realized this mistake and published these documents unedited on the 22nd of March 2018, after data were collected and partly analyzed.

2 https://osf.io/57nm8/

3 https://osf.io/2cnrf/

4 As compared to the pre-registration, we used the terms purity and harm in place of sanctity and care to refer to the same types of moral transgressions. We did so to be more consistent with the terminology used in the literature.
